# Editorial: Antimicrobial peptides and their druggability, bio-safety, stability, and resistance

**DOI:** 10.3389/fmicb.2024.1425952

**Published:** 2024-05-23

**Authors:** Xuanxuan Ma, Rustam Aminov, Octavio Luiz Franco, Cesar de la Fuente-Nunez, Guangshun Wang, Jianhua Wang

**Affiliations:** ^1^Innovative Team of Antimicrobial Peptides and Alternatives to Antibiotics, Feed Research Institute, Chinese Academy of Agricultural Sciences, Beijing, China; ^2^Gene Engineering Laboratory, Feed Research Institute, Chinese Academy of Agricultural Sciences, Beijing, China; ^3^Key Laboratory of Feed Biotechnology, Ministry of Agriculture and Rural Affairs, Beijing, China; ^4^The School of Medicine, Medical Sciences and Nutrition, University of Aberdeen, Aberdeen, United Kingdom; ^5^S-Inova Biotech, Universidade Católica Dom Bosco, Campo Grande, MS, Brazil; ^6^Centro de Análises Proteômicas e Bioquímicas Programa de Pós-Graduação em Ciências Genômicas e Biotecnologia, Universidade Católica de Brasília, Brasília, DF, Brazil; ^7^Machine Biology Group, Departments of Psychiatry and Microbiology, Perelman School of Medicine, Institute for Biomedical Informatics, Institute for Translational Medicine and Therapeutics, University of Pennsylvania, Philadelphia, PA, United States; ^8^Departments of Bioengineering and Chemical and Biomolecular Engineering, School of Engineering and Applied Science, University of Pennsylvania, Philadelphia, PA, United States; ^9^Department of Chemistry, School of Arts and Sciences, University of Pennsylvania, Philadelphia, PA, United States; ^10^Penn Institute for Computational Science, University of Pennsylvania, Philadelphia, PA, United States; ^11^Department of Pathology, Microbiology, and Immunology, University of Nebraska Medical Center, Omaha, NE, United States

**Keywords:** antimicrobial peptide (AMPs), druggability, bio-safety, stability, resistance

## 1 Introduction

The excessive and often indiscriminate use of antibiotics in many areas of human activities has caused a widespread antibiotic resistance, which poses a major threat to the public health worldwide (Carratalá et al., 2020; Murray et al., 2022; Bessa et al., 2023; De la Fuente-Núñez et al., 2023). Even more worrying is the dearth of new antimicrobial drugs (Durand et al., 2019; Li S. et al., 2021). Under these circumstances, the development of new antimicrobial drugs is essential (Tacconelli et al., 2018; Hamad et al., 2019). Antimicrobial peptides (AMPs) have attracted attention for their potent antibacterial activities and unique antibacterial mechanisms, which are efficient against many bacterial pathogens, including those that are multidrug-resistant (MDR) (Boaro et al., 2023; Maasch et al., 2023; Wong et al., 2023; Xuan et al., 2023). However, the entry of AMPs into clinical practice has encountered many challenges, including peptide stability, bioavailability, and toxicity, all of which limit their clinical applicability (Durand et al., 2019; Sarkar et al., 2021). Therefore, rational design, advanced drug formulations and tailored routes of administration and delivery systems are crucial for the development of AMPs as viable therapeutic options. The third volume of the Research Topic on AMPs targeted the above issues to bring AMPs closer to clinical practice.

## 2 Challenges in the clinical translation of AMPs

### 2.1 Low bioavailability *in vivo*

Despite the intrinsic properties of AMPs that make them highly attractive for a potential use, relatively few of them have been successfully translated into the clinical use or as food preservatives (Mishra et al., 2017; Costa et al., 2019; Adaro et al.; Koniuchovaitė et al.). One of the key constraints is the mismatch between their *in vivo* and *in vitro* activities. Particularly frustrating is the fact that highly anticipated peptides such as pexiganan, iseganan, neuprex and omiganan have failed in phase III clinical trials due to low *in vivo* efficacy (http://dramp.cpu-bioinfor.org/). Many factors may contribute to the low bioavailability *in vivo*. However, poor stability of these molecules in complex microenvironments has been identified as the most significant factor (Jiang et al., 2021; Fu et al.; Guevara-Lora et al.; Skłodowski et al.).

### 2.2 Toxicity

One of the important prerequisites for clinical use is the drug safety, and this is the second major obstacle on the way of AMPs toward clinical translation (Payne et al., 2015). Toxicity of AMPs includes cytotoxicity and systemic toxicity (Li et al., 2017). Cytotoxicity is usually an inherent property of membrane-active AMPs, the cationic and hydrophobic components of which can directly interact with the membrane of host cells (Agrillo et al.), This interaction is exhibited in a concentration-dependent toxicity. Typical examples are melittin, CZS-1 and alamethicin, which exhibit potent cytotoxicity, including hemolysis (Askari et al., 2021; Farid et al., 2023; Bermúdez-Puga et al.; Brakel et al.). Considering the potential cytotoxic mechanisms of AMPs relative to their successful application, it can be generally concluded that narrow-spectrum peptides are relatively safer for clinical translation due to their lower cytotoxicity and the lack of off-target effects against the beneficial microbiota (Xu et al., 2020; Zong et al., 2020). Conversely, broad-spectrum AMPs tend to display the increased cytotoxicity toward the host and adverse effects on the microbiota, thereby limiting their potential for clinical use (Hao et al., 2023). Systemic toxicity may result from off-target effects, accumulation of drug in kidneys, undesirable immune responses or chronic inflammation (e.g., atopic dermatitis or hidradenitis suppurativa) due to the increased drug concentrations (Takahashi et al., 2018). Therefore, preclinical safety evaluation of AMPs should not be limited to basic hemolysis and cytotoxicity but also requires the evaluation of systemic toxicity. In fact, the antimicrobial and immunomodulatory properties and toxicity of AMPs are often compounded. Thus, a careful attention has to be paid to the delicate balance of antimicrobial properties, immunomodulation, and toxicity.

### 2.3 Pharmacokinetic assays

Although several papers in this Research Topic have discussed the pharmacokinetic (PK) properties of AMPs, it has to be emphasized here that PK is still a bottleneck for AMP translation. It is known that the physicochemical properties of AMPs are quite different from the traditional small-molecule chemical drugs. Hence, the PK of traditional small-molecule drugs should be further modified, improved and optimized for AMPs so that the quantitative PK methodology can be successfully applied for this class of antimicrobials (Wang et al., 2012). Therefore, the development of suitable quantification methods for PK of AMPs, which are different from small-molecule chemical drugs, is the 3rd key challenge for their entry into clinical applications (Ewles and Goodwin, 2011; Mercer and O'Neil, 2013). Usually, linear cationic AMPs are rapidly metabolized *in vivo* and degraded into smaller fragments or amino acids and absorbed as nutrients. This process interferes with the determination of the main four PK parameters such as absorption, distribution, metabolism, and excretion. Although the safety of AMP degradation products, especially amino acids, *in vivo* is not of a major concern from the nutrient metabolism point of view, it is difficult to determine the concentration of these products with the use of regular analytical tools. Therefore, there is an urgent need for updating PK principles so that they suit to AMPs, especially protocols for their clinical evaluation (Giguère et al., 2017). In brief, we believe that the use of the latest material analysis methods for exploratory pharmacokinetic detection combined with the calculation of PK parameters based on non-compartmental model is an important prerequisite for AMPs to resolve the bottleneck of drug development and transition to clinical practice (Zheng et al., 2022, 2024).

### 2.4 Resistance

The likelihood of resistance development toward AMPs is generally much lower than that against conventional antibiotics. Numerous parameters influence resistance development, including the dose used, period of application, temperature, exposure/contact with inhibitory substances, and others. Metabolic pathways and genes within bacterial cells can be replaced or compensated over time, as has been shown for defensins derived from plants and polymyxin from microbes (Ouyang et al.); On the contrary, molecules that have multiple targets in bacteria are less likely to select for bacterial resistance. AMPs with the low probability of resistance development include melittin, bombesin, venoms and cecropins (Chen et al., 2022a). Additional attention has to be paid to AMP-induced cross-resistance. Chen et al. (2022b) found that *Staphylococcus aureus* acquired limited resistance to PIS-3, with a concomitant resistance toward polymyxin B, vancomycin, and tetracycline, but with no resistance development toward PIS-1. Thus, it is important to gain a better understanding of pharmacology, evolutionary effects and potential resistance acquisition during the development and application of AMPs, the above steps have been largely ignored in the past with traditional antibiotics (Lazzaro et al., 2020).

## 3 R&D directions of AMPs

### 3.1 AMP stabilization technology

The molecular stability of AMPs is another important parameter to take into account. The stability of these agents needs to be sufficient to exert their function, ideally without causing off-target effects. At the same time, when assessing peptide stability, it is necessary to focus on the route of administration as this may substantially affect stability.

#### 3.1.1 Chemical modification

Strategies for the improvement of stability of AMPs include two complementary approaches. The first is chemical modification(s) to improve the stability and bioavailability and reduce toxicity. Currently effective chemical modifications include the following:

1. Replacement of L-amino acids in natural sequences with proteinogenic amino acids (unnatural α-amino acids, unnatural β-amino acids, unnatural γ-amino acids, and D-amino acids) (De la Fuente-Núñez et al., 2015; Zhang et al., 2016; Sandín et al., 2021; He et al., 2023). For example, Li T. et al. (2021) used D-amino acids (Val and Pro) to replace the natural L-amino acids in N6 to improve the stability of the antibacterial N6NH2 against protease.
2. Cyclisation is an effective strategy to improve the metabolic stability of AMPs. This notion is supported by the fact that some of the successfully marketed AMPs are cyclic such as bacitracin A, daptomycin, polymyxins B1 and B2 (Falanga et al., 2017; Mishra et al., 2017; Costa et al., 2019; Liu et al.).
3. PEG modification is one of the effective methods to improve the biocompatibility and bioavailability of peptides. The success of this approach was proven in a number of studies, involving AMPs such as OM19r-8, N6NH_2_ and SAMP-A_4_, the stability of which was substantially improved by PEG modification (Lau and Dunn, 2018; Manteghi et al., 2020; Li R. et al., 2021; Li et al., 2022).
4. N-/C-terminal modification (C-terminal amidation, N-terminal acylation or methylation modification) is the most straightforward methods to improve the AMP stability (Teixeira et al., 2010; Li D. et al., 2021). Although these AMP modification methods have been supported by several corresponding studies, they are not universal and each peptide may require a set of their own design strategies depending on the peptide scaffold and the desired activity (Torres et al., 2018, 2019; Silva et al., 2020; Cesaro et al., 2022).

#### 3.1.2 Delivery systems

In addition to chemical modifications, improvements in pharmacokinetics and pharmacodynamics of AMPs can be achieved via the use of nanotechnology, which may increase the stability of AMPs and thus facilitate their clinical translation (Carratalá et al., 2020; Cesaro et al., 2023; Xuan et al., 2023). Currently, various types of carriers are employed in AMP delivery studies (Li et al., 2023):

1. These can be inorganic materials such as mesoporous silica, metal nanoparticles, carbon nanotubes, and others. Izquierdo-Barba et al. (2009), for instance, demonstrated that incorporation of antimicrobial peptide LL-37 into mesoporous silica significantly increased its half-life, with the maximum release rate of LL-37 achieved after 200 h.
2. Organic polymers such as chitosan, polylactide-glycolide (PLGA), liposomes and others can also serve as efficient delivery systems for AMPs. For example, d'Angelo et al. (2015) demonstrated that chitosan and PLGA-coated colistin could be continuously released in biofilms, thereby eradicating biofilms formed by *Pseudomonas aeruginosa*. In another study, Ma et al. (2024) successfully increased the trypsin tolerance of AMP NZ2114 by 4.24-fold using PLGA encapsulation.
3. Another approach to improve the pharmacokinetics and pharmacodynamics of AMPs is the use of peptide self-assembly properties. As comprehensively overviewed by Habibi et al. (2016) and Zou et al. (2020), a variety of peptides can self-assemble into nanoparticles, nanofibers or nanogels according to their hydrophobicity, length, and structures to achieve precisely controlled release rates. Self-assembly of peptides can also overcome the problem of low encapsulation efficiency and release rates of traditional coating strategies. This approach, therefore, has attracted a great interest for potential applications in drug delivery, functional materials, and regenerative medicine. Recently, an increasing number of studies have supported the view that self-assembly of AMPs can effectively increase their stability, prolong the half-life and improve biosafety, thus contributing to better pharmacokinetic and pharmacodynamic properties of AMPs (Chen et al., 2019; Tram et al., 2022).

### 3.2 AMP application strategy

After exploration toward clinical translation for over 50 years, there is a huge number of publications and patents with innovative results on AMPs, but also there is still room for improvement, and it is expected that the original intentions could be realized as soon as possible (Zasloff, 2015; Czaplewski et al., 2016; Arciola et al., 2018).

#### 3.2.1 Topical applications

Among the 11 commercially available AMPs, daptomycin, dalbavancin, telavancin and oritavancin were initially approved for the treatment of skin infections, bacitracin and polymyxin B—for conjunctivitis and keratitis, and tyrothricin—for acute pharyngitis. Besides, the majority of AMP drugs currently in clinical trials are intended for topical use (http://dramp.cpu-bioinfor.org/). The emphasis of pharmaceutical companies on topical AMP drugs is logical and economically feasible because topical administration does not require the level of pharmacokinetic and pharmacodynamic characterization required for the internal use. An increasing number of studies have shown that AMPs play a crucial role in promoting wound infection clearance and recovery (Gao et al., 2023) and in managing local inflammation in pyoderma, conjunctivitis, mastitis, and biofilms (Yang et al., 2022; Zhang et al., 2024; Fernández-Fernández et al.; Ji et al.; Jiang et al.). Thus the topical use of AMPs asserts their promising prospects as a viable treatment option.

#### 3.2.2 Drug combinations

Antibiotic combinations became important therapeutic tools to deal with multidrug-resistant or mixed infections. Other advantages include synergetic effects between antibiotics that allow the decrease of antibiotic concentration(s), especially of toxic ones, and also a lower probability of resistance development. In this regard, combination of AMPs with traditional antibiotics is also a valuable approach (Reffuveille et al., 2014; Mishra et al., 2017; Mhlongo et al., 2023; Chen X. et al.). AMPs, antibiotics and vaccines could complement each other to maintain the health of the organism (Hao et al., 2022; Yang et al., 2023), these combination therapies can improve both the efficacy of treatment and reduce the dose of each drug, thereby reducing excessive toxicity and side effects, while maintaining a reasonable balance between the therapeutic efficiency and drug resistance development (Zakaryan et al., 2021). For example, the combination of AMP OM19r with gentamicin increased the antibacterial activity of the latter against MDR *Escherichia coli* B2 by 64-fold (Cui et al.). Thus, AMPs can increase the permeability of the cytoplasmic membrane, which facilitates the entry of antibiotics into bacterial cells (Duong et al., 2021). The combination of cecropin D-derived peptide and caspofungin showed the synergistic effects against *Candida albicans* (Guevara-Lora et al.). The study of Alencar-Silva et al. (2023) demonstrated the decreased cytotoxicity of Synoeca MP through its combination with IDR-1018. The combination also enhanced cell proliferation and migration and accelerated wound re-epithelialization, which opens the possibility for the development of new strategies in treatment of skin injuries (Alencar-Silva et al., 2023).

Presently the Antimicrobial Peptide Database (APD) (https://aps.unmc.edu) contains the information about 4231 peptides, from which 3223 are natural AMPs. The use of the majority of them is limited to topical and combination applications, including the early or preventive treatments. Thus, there are decreasing numbers of cases, where treatments involving AMPs include emergency treatments at ICUs or treatment of serious infections in modern husbandry.

### 3.3 Reduction of AMPs production costs

Two main AMPs production routes include chemical synthesis and recombinant expression. Chemical synthesis can be executed via solid and liquid phase synthesis methods or their combination. The representative examples of peptide-based drug production at multi-ton scale are HIV fusion inhibitory peptide T-20 (Fuzeon, Roche), semaglutide and insulin (Walsh, 2005; Thayer, 2011; Aggarwal et al., 2021). With technological advances, more and more AMPs including those longer than 30 amino acids, with complex structures and modification processes, will be industrialized utilizing chemical synthesis or transgenic expressions at acceptable costs. Multiple studies have been published recently along this line. For example, optimisation of culture conditions of recombinant *Pichia pastoris* and induction process of cathelicidin BF expression allowed to reach the product concentration of 0.5 g/L after 240 h (Dong et al.). Li and Chen engineered synthetase to create a synthetic pathway for the production of a novel fusaricidin and constructed the recombinant M6 yielding a 55 mg/L of fusaricidin LI-F07a. In addition, the codon use optimisation in heterologous expression of AFP in *E. coli* allowed to reach its production at 780 μg/ml (Chen Y.-P. et al.). Different strategies have been used to optimize AMPs production and develop large-scale facilities, some of which were successfully accomplished. For example, after nearly two decades of efforts, Wang's team has successfully established a 20 and 30 cubic meter-scale production system for high-yield preparation of plectasin analogs with an affordable cost comparable to traditional antibiotics, a milestone for the translation of AMPs (Zhang et al., 2014; Yang et al., 2022; Hao et al., 2023; Jin et al., 2023; Li et al.).

## 4 Conclusions

Based on their antimicrobial activities and immunomodulatory properties, AMPs can be used for disease prevention and treatment. However, the development of AMPs as a viable therapeutic option faces challenges such as cytotoxicity, stability, and bioavailability. As discussed below, the strategies for the development of different AMP classes may require their own specific challenges to be addressed.

**Figure 1:**
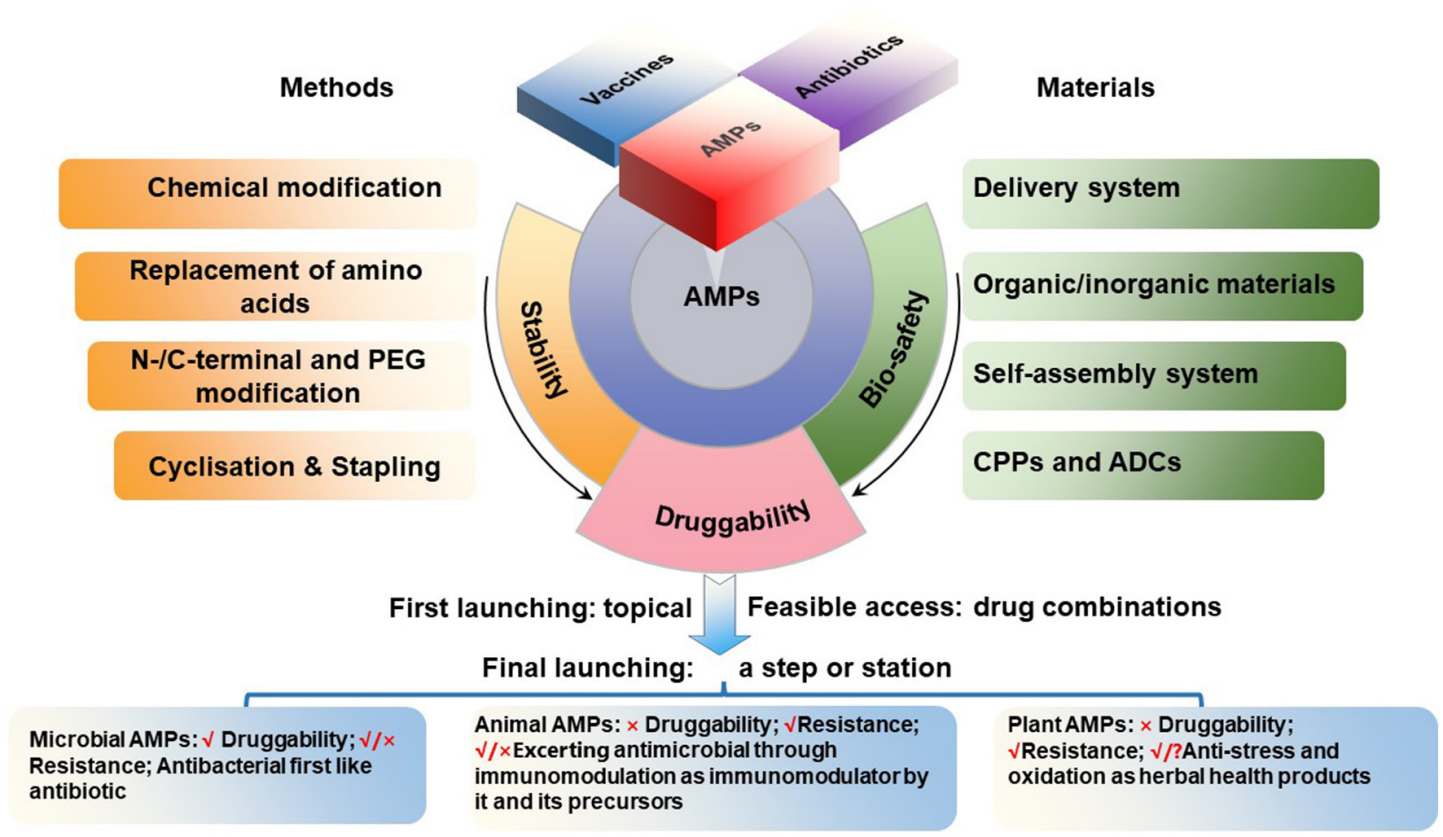
Roadmap for AMP development.

1. The category of microbial AMPs is very broad, covering natural AMPs from four kingdoms of life (bacteria, archaea, protists, and fungi) as annotated in APD (Wang et al., 2016; Santos-Júnior et al., 2023; Wang, 2023; https://aps.unmc.edu). Most of them share natural druggability properties similar to traditional microbial antibiotics. Microbial AMPs are made either ribosomally or non-ribosomally. Ribosomally synthesized peptides are exemplified by nosiheptide, nisin, plectasin and its derivatives, while non-ribosomally synthesized peptides are represented by vancomycin, polymyxins and daptomycin, all currently in medical use. Thus, these AMPs can be easily developed by following the path of modern antibiotic pharmaceutical industry, including genetic modification, recombinant expression and chemical synthesis. These AMPs are expected to have a wide range of therapeutic uses. A potential drug resistance emergence, however, should be carefully monitored during the drug development process as well as during the use. At same time, need to keep in mind the similarity and differences in their dual transmembrane entry mechanisms and targets/paths, which should be separately addressed in animals and pathogens (Figure 1).
2. After the recent refinement, the category of animal AMPs in the APD include 2515 representatives from both invertebrates and vertebrates. These include melittin, bombesin, venoms, and cecropins with weak druggability, because their strong antimicrobial activity is accompanied by high toxicity and interference with immunological and metabolic processes. Their development, therefore, has been more difficult and complicated. There are possibilities that these peptides or their derivatives could be developed as antiviral (Guo et al.) or anti-cancer (Qu et al.) drugs. Nevertheless, some animal AMPs such as small and cyclic θ-defensins possess a great development potential as antibacterial agents (Schaal et al., 2021), as demonstrated for cathelicidin-derived PAM-1 against ceftazidime-avibactam (CZA)-resistant *E. coli*. (Han et al.). Additional mechanistic studies are necessary for better understanding whether the impact of AMPs *in vivo* is a consequence of immunological or other metabolic regulation or brakue to the bona fide antimicrobial activity (Figure 1).
3. After the recent refinement, the category of plant AMPs in the APD includes 258 plant peptides with known antimicrobial activities. Compared to the first two categories, the number of AMPs in this category is relatively less and include peptide compounds such as plant defensins (for instance, Rs-AFP1), thionins (for instance, Tu-AMP 1), soybean peptides and other peptide products that can be developed into products with antimicrobial and other activities (Shwaiki et al., 2021; Sharma et al., 2022). Many traditional herbal medicines in China and other countries have been used for millennia as anti-infective agents. In the modern medicine, however, the active antimicrobial compounds have to be purified, characterized, and thus their mechanism(s) of action must be elucidated and revealed. In this regard, traditional herbal medicines represent a valuable source of potentially useful AMPs, which can be explored further for our benefit (Figure 1).

The current structure-and-function and spatiotemporal relationship of AMPs is the product of long-term evolution of these molecules, with the selection of molecules that provided the best protection of host organisms against the invasion of other organisms, mainly microorganisms. The use of these molecules by humans in medicine or in other applications not necessarily coincides exactly with the functions that have been selected during the previous natural evolutionary process. For example, AMPs are continuously produced by living organisms and act *in situ*, while humans need them in various acceptable pharmaceutical formulations, with the concomitant problem of stability or bioavailability. Thus, our task in this Research Topic was to provide a framework for future development of AMPs for the use in medicine and other applications (Figure 1). We hope this Research Topic of 22 papers contributed to this goal (Supplementary Table S1).

## Author contributions

XM: Visualization, Writing – original draft, Data curation, Formal analysis, Investigation, Validation. RA: Conceptualization, Methodology, Resources, Supervision, Writing – review & editing, Investigation, Formal analysis. OF: Supervision, Writing – review & editing. CF-N: Conceptualization, Funding acquisition, Supervision, Writing – review & editing. GW: Conceptualization, Funding acquisition, Software, Supervision, Writing – review & editing. JW: Conceptualization, Funding acquisition, Methodology, Resources, Supervision, Writing – original draft, Writing – review & editing.
