# Supplementary material for: Editorial: Antimicrobial peptides and their druggability, bio-safety, stability, and resistance
Source: Front Microbiol. 2024 May 23;15:1425952. doi: 10.3389/fmicb.2024.1425952 (PMC11154904; doi:10.3389/fmicb.2024.1425952)
Supplement: Supplementary file 1 [file Table_1.docx]

**Editorial:** **Antimicrobial Peptides: Druggability, Bio-safety, Stability, and Resistance**

Xuanxuan Ma ^1, 2, 3^, Rustam Aminov ^4*^, Octavio Luiz Franco ^5, 6*^, Cesar de la Fuente-Nunez ^7, 8, 9, 10*^, Guangshun Wang ^11*^ and Jianhua Wang ^1, 2, 3*^

^1^Innovative Team of Antimicrobial Peptides and Alternatives to Antibiotics, Feed Research Institute, Chinese Academy of Agricultural Sciences, Beijing, China

^2^Gene Engineering Laboratory, Feed Research Institute, Chinese Academy of Agricultural Sciences, Beijing, China

^3^Key Laboratory of Feed Biotechnology, Ministry of Agriculture and Rural Affairs, Beijing, China

^4^The School of Medicine, Medical Sciences and Nutrition, University of Aberdeen, Aberdeen, United Kingdom

^5^S-Inova Biotech, Universidade Católica Dom Bosco, Campo Grande, MS, Brazil

^6^Centro de Análises Proteômicas e Bioquímicas Programa de Pós-Graduação em Ciências Genômicas e Biotecnologia, Universidade Católica de Brasília, Brasília, DF, Brazil

^7^Machine Biology Group, Departments of Psychiatry and Microbiology, Institute for Biomedical Informatics, Institute for Translational Medicine and Therapeutics, Perelman School of Medicine, University of Pennsylvania, Philadelphia, PA, United States

^8^Departments of Bioengineering and Chemical and Biomolecular Engineering, School of Engineering and Applied Science, University of Pennsylvania, Philadelphia, PA, United States

^9^Department of Chemistry, School of Arts and Sciences, University of Pennsylvania, Philadelphia, PA, United Sates of America.

^10^Penn Institute for Computational Science, University of Pennsylvania, Philadelphia, PA, United States

^11^Department of Pathology, Microbiology, and Immunology, University of Nebraska Medical Center, Omaha, NE, United States

*Correspondence: Jianhua Wang: wangjianhua@caas.cn; wangjianhua.peking@qq.com; Rustam Aminov: rustam.aminov@gmail.com; Octavio Luiz Franco: ocfranco@gmail.com; Cesar de la Fuente-Nunez: cfuente@upenn.edu; Guangshun Wang: gwang@unmc.edu

**Keywords**: antimicrobial peptides (AMPs), druggability, bio-safety, stability, resistance

Table S1. Distribution of 22 papers as citation in this issue

| Section | The cited issue articles |
| --- | --- |
| 1.1/2.2.2 | Guevara-Lora I, et al. Cecropin D-derived synthetic peptides in the fight against *Candida albicans* cell filamentation and biofilm formation. Front Microbiol. 2023; 13: 1045984. doi:10.3389/fmicb.2022.1045984 |
| 1.1 | Fu Q, et al. Prediction and bioactivity of small-molecule antimicrobial peptides from *Protaetia brevitarsis* Lewis larvae. Front Microbiol. 2023; 14: 1124672. doi:10.3389/fmicb.2023.1124672 |
| 1.1 | Skłodowski K, et al. Ceragenins exhibit bactericidal properties that are independent of the ionic strength in the environment mimicking cystic fibrosis sputum. Front Microbiol. 2023; 14: 1290952. doi:10.3389/fmicb.2023.1290952 |
| 1.1 | Koniuchovaitė A, Petkevičiūtė A, Bernotaitė E, et al. Novel leaderless bacteriocin geobacillin 6 from thermophilic bacterium *Parageobacillus thermoglucosidasius*. Front Microbiol. 2023;14:1207367. Published 2023 Jun 15. doi:10.3389/fmicb.2023.1207367 |
| 1.1 | Adaro M, Ibáñez ÁGS, Origone AL, et al. Enzymatic synthesis of new antimicrobial peptides for food purposes. Front Microbiol. 2023;14:1153135. Published 2023 May 16. doi:10.3389/fmicb.2023.1153135 |
| 1.2 | Agrillo B, et al. Antimicrobial activity, membrane interaction and structural features of short arginine-rich antimicrobial peptides. Front Microbiol. 2023; 14: 1244325. doi:10.3389/fmicb.2023.1244325 |
| 1.2 | Bermúdez-Puga S, et al. Dual antibacterial mechanism of [K4K15] CZS-1 against *Salmonella Typhimurium*: a membrane active and intracellular-targeting antimicrobial peptide. Front Microbiol. 2023; 14: 1320154. Published 2023 Dec 14. doi:10.3389/fmicb.2023.1320154 |
| 1.2 | Brakel A, et al. Evaluation of proline-rich antimicrobial peptides as potential lead structures for novel antimycotics against *Cryptococcus neoformans*. Front Microbiol. 2024; 14: 1328890. doi:10.3389/fmicb.2023.1328890 |
| 1.4 | Ouyang Z, et al. Mechanistic and biophysical characterization of polymyxin resistance response regulator PmrA in *Acinetobacter baumannii*. Front Microbiol. 2024; 15: 1293990. Published 2024 Feb 27. doi:10.3389/fmicb.2024.1293990 |
| 2.1.1 | Liu SX, et al. Recent advances on cyclodepsipeptides: biologically active compounds for drug research. Front Microbiol. 2023; 14: 1276928. doi:10.3389/fmicb.2023.1276928 |
| 2.2.1 | Fernández-Fernández R, et al. Detection of antimicrobial producing*Staphylococcus* from migratory birds: Potential role in nasotracheal microbiota modulation. Front Microbiol. 2023; 14: 1144975. doi:10.3389/fmicb.2023.1144975 |
| 2.2.1 | Ji F, et al. Antimicrobial peptide 2K4L disrupts the membrane of multidrug-resistant *Acinetobacter baumannii*and protects mice against sepsis. Front Microbiol. 2023; 14: 1258469. Published 2023 Oct 24. doi:10.3389/fmicb.2023.1258469 |
| 2.2.1 | Jiang S, et al. Antimicrobial peptide temporin derivatives inhibit biofilm formation and virulence factor expression of *Streptococcus mutans*. Front Microbiol. 2023; 14: 1267389. doi:10.3389/fmicb.2023.1267389 |
| 2.2.2 | Chen X, et al. Anti-Pseudomonas aeruginosa activity of natural antimicrobial peptides when used alone or in combination with antibiotics. Front Microbiol. 2023; 14: 1239540. Published 2023 Sep 5. doi:10.3389/fmicb.2023.1239540 |
| 2.2.2 | Cui Q, et al. Antibiotic synergist OM19r reverses aminoglycoside resistance in multidrug-resistant *Escherichia coli*. Front Microbiol. 2023; 14: 1144946. doi:10.3389/fmicb.2023.1144946 |
| 2.3 | Li Y, et al. Structure modification of an antibiotic: by engineering the fusaricidin bio-synthetase A in *Paenibacillus polymyxa*. Front Microbiol. 2023; 14: 1239958. doi:10.3389/fmicb.2023.1239958 |
| 2.3 | Dong X, et al. High expression of antimicrobial peptides cathelicidin-BF in *Pichia pastoris* and verification of its activity. Front Microbiol. 2023; 14: 1153365. Published 2023 Jun 9. doi:10.3389/fmicb.2023.1153365 |
| 2.3 | Chen YP, et al. Characterization and expression of fungal defensin in *Escherichia coli*and its antifungal mechanism by RNA-seq analysis. Front Microbiol. 2023; 14: 1172257. doi:10.3389/fmicb.2023.1172257 |
| 2.3 | Li X, et al. Plectasin: from evolution to truncation, expression, and better druggability. Front Microbiol. 2023; 14: 1304825. doi:10.3389/fmicb.2023.1304825 |
| 3.2 | Guo X, et al. Cathelicidin-derived antiviral peptide inhibits herpes simplex virus 1 infection [published correction appears in Front Microbiol. 2023 Jul 25; 14: 1254775]. Front Microbiol. 2023; 14: 1201505. doi:10.3389/fmicb.2023.1201505 |
| 3.2 | Qu B, et al. Anticancer activities of natural antimicrobial peptides from animals. Front Microbiol. 2024; 14: 1321386. doi:10.3389/fmicb.2023.1321386 |
| 3.2 | Han Y et al., PAM-1: An antimicrobial peptide with promise against ceftazidimeavibactam resistant *Escherichia coli* infection. Front Microbiol. 2024; 15: 1291876. doi.org/10.3389/fmicb.2024.1291876 |

**Author contributions** The first draft text of this editorial was written by XM as assistant of JW and his co-editors with their guide and direction. All authors contributed to the article and approved the submitted version.

**Funding** This work was supported by the National Natural Science Foundation of China (Grants No. 31872393, 2018–2022), National Key R&D Plan - High Expression of Thiopeptides and their Analogs (2022YFC2105000-03, 2022–2026), the National Agricultural Science and Technology Innovation Program (ASTIP) of Chinese Academy of Agricultural Sciences (CAAS-ASTIP-2017-FRI-02, 2013), and its key projects of Alternatives to Antibiotics for Animal (Grant No. CAAS-ZDRW202111, 2021–2023) and Feed (Grant No. CAASZDXT2018008, 2018–2020) Usages. OF was supported by CAPES, CNPq, FAPDF, and FUNDECT. CF-N was supported by the AIChE Foundation, a BBRF Young Investigator Grant, the Dean’s Innovation Fund of the Perelman School of Medicine at the University of Pennsylvania, and other funds from the National Institute of General Medical Sciences of the National Institutes of Health (R35GM138201) and the Defense Threat Reduction Agency (DTRA; HDTRA11810041 and HDTRA1-21-1-0014). GW was supported by funds from National Institute of General Medical Sciences (R01GM138552) and National Institute of Allergy and Infectious Diseases (R56AI175209), the National Institutes of Health, USA as well as Chair, Department of Pathology, Microbiology, and Immunology of the University of Nebraska Medical Center (Omaha, Nebraska, USA) for the cost of maintenance of the Antimicrobial Peptide Database (<https://aps.unmc.edu>). This manuscript documents the opinions of the authors/editors and does not represent the funding agencies.

**Acknowledgments** We would like to sincerely thank a total of 192 authors of 23 papers and over 80 peer editors and reviewers for their valuable professional contributions into this Research Topic Antimicrobial Peptides and their Druggability, Bio-safety, Stability, and Resistance, along with the staff of Frontiers in Microbiology, and also team supports of five Topic editors JW, RA, OF, CF-N and GW.

**Conflict of interest:**

OF was employed by S-Inova Biotech.

The remaining authors declare that the research was conducted in the absence of any commercial or financial relationships that could be construed as a potential conflict of interest.

The author(s) declared that they were an editorial board member of Frontiers, at the time of submission. This had no impact on the peer review process and the final decision.

**Publisher’s note**: All claims expressed in this article are solely those of the authors and do not necessarily represent those of their affiliated organizations, or those of the publisher, the editors and the reviewers. Any product that may be evaluated in this article, or claim that may be made by its manufacturer, is not guaranteed or endorsed by the publisher.

(END)
